# Metabolic Reprogramming in Glioblastoma Multiforme: A Review of Pathways and Therapeutic Targets

**DOI:** 10.3390/cells13181574

**Published:** 2024-09-19

**Authors:** Ashley Irin Cortes Ballen, Maryam Amosu, Surya Ravinder, Joey Chan, Emre Derin, Hasan Slika, Betty Tyler

**Affiliations:** Hunterian Neurosurgical Laboratory, Department of Neurosurgery, The Johns Hopkins University School of Medicine, Baltimore, MD 21231, USA; acortes3@jhu.edu (A.I.C.B.); mamosu1@jhu.edu (M.A.); sravind2@jhu.edu (S.R.); jchan58@jhu.edu (J.C.); ederin2@jhmi.edu (E.D.); hslika1@jhmi.edu (H.S.)

**Keywords:** glioblastoma multiforme, metabolic reprogramming, tumor microenvironment, Warburg effect, glycolysis, therapeutic drugs

## Abstract

Glioblastoma (GBM) is an aggressive and highly malignant primary brain tumor characterized by rapid growth and a poor prognosis for patients. Despite advancements in treatment, the median survival time for GBM patients remains low. One of the crucial challenges in understanding and treating GBMs involves its remarkable cellular heterogeneity and adaptability. Central to the survival and proliferation of GBM cells is their ability to undergo metabolic reprogramming. Metabolic reprogramming is a process that allows cancer cells to alter their metabolism to meet the increased demands of rapid growth and to survive in the often oxygen- and nutrient-deficient tumor microenvironment. These changes in metabolism include the Warburg effect, alterations in several key metabolic pathways including glutamine metabolism, fatty acid synthesis, and the tricarboxylic acid (TCA) cycle, increased uptake and utilization of glutamine, and more. Despite the complexity and adaptability of GBM metabolism, a deeper understanding of its metabolic reprogramming offers hope for developing more effective therapeutic interventions against GBMs.

## 1. Introduction

Glioblastoma (GBM) is one of the most aggressive central nervous system tumors and is the predominant malignant tumor originating in the brain [1]. According to The Central Brain Tumor Registry of the United States (CBTRUS), GBMs account for around 14.2% of all CNS tumors and 50.9% of all malignant brain tumors [2]. The current management of GBMs consists of achieving maximal safe surgical removal of the tumor with adjuvant chemoradiotherapy. In addition, innovative strategies and therapies are continuously being investigated for the treatment of GBMs, including CAR-T cell therapies that target two main proteins commonly found on tumor cells [3]. Despite these advances, the outlook for patients continues to be challenging, with a 5-year overall survival rate of less than 7% [2]. This calls for the need to understand the underlying mechanisms that drive the tumor’s ability to proliferate and invade, even under harsh conditions such as hypoxia. Under such conditions, tumor cells must reprogram their metabolic pathways to meet the requirements of rapid proliferation and resistance to cell death, a process known as metabolic reprogramming. This reprogramming does not only serve as an adaptive mechanism to keep up with the tumor’s increasing demand for biosynthetic molecules and energy, but it also leads to the generation of metabolites that are implicated in different oncogenic pathways [4]. This review explores the extensive metabolic reprogramming paradigms in glioblastomas. Altered metabolic pathways, such as the pentose phosphate pathway, are highlighted to elucidate the GBM’s reliance on glycolysis over oxidative phosphorylation. Amino acid metabolism is also discussed while emphasizing glutamine’s role in GBM proliferation and invasion. Lipid metabolism is tackled, with a focus on altered synthesis and uptake of lipids that support GBM’s growth in hypoxic conditions. This review further delves into nucleotide metabolism, the TCA cycle, and oxidative phosphorylation, further describing GBM’s metabolic adaptations. Finally, potential targets within these metabolic pathways are identified, offering insights into future therapeutic options. We will also discuss the primary drivers of these metabolic changes, such as hypoxia, the tumor microenvironment, immune system interactions, and genetic and epigenetic factors. Subsequently, we will build on that to highlight strategies that target these metabolic pathways to enhance treatment efficacy and patient survival.

## 2. Rewired Metabolic Pathways in GBM

### 2.1. Glycolysis (Warburg Effect) and Pentose Phosphate Pathway (PPP)

**Figure 1:**
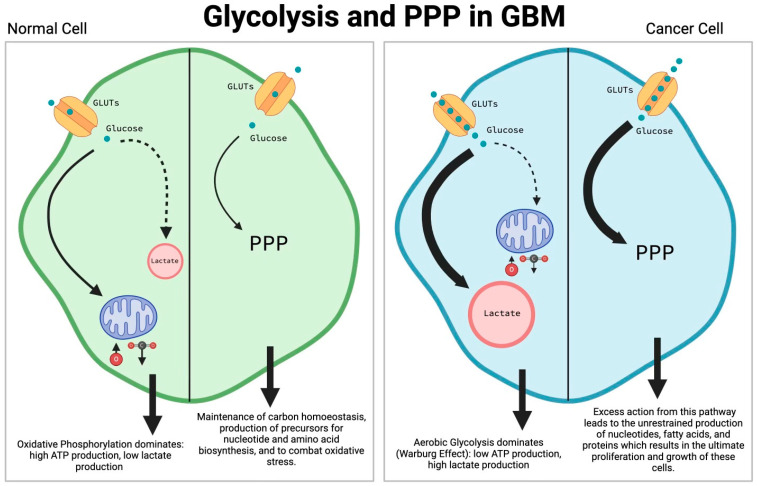
The alteration of glycolysis and the PPP in GBM. In healthy cells, oxidative phosphorylation is the dominant pathway, resulting in higher ATP production and low intercellular lactate concentrations. In tumor cells, a phenomenon called the Warburg effect highlights the preference of tumor cells to take up increased amounts of glucose and produce lactate regardless of the presence of oxygen (aerobic glycolysis). The PPP in normal cells operates at a homeostatic rate, utilizing glucose to produce compounds important for nucleotide and amino acid creation. In GBM tumor cells, the pathway is excessively active, producing nucleotide precursors that are useful downstream mechanisms for increasing the size and number of tumor cells. The rewiring of glycolysis and the PPP combined are critical to the survival and further proliferation of cancer cells. ATP, adenosine triphosphate; GBM, glioblastoma multiforme; PPP, pentose phosphate pathway.

Glucose serves as an important nutrient for the brain, fueling functionally critical roles including the production of adenosine triphosphate (ATP), neurotransmitter synthesis, oxidative stress management, and other crucial functions [5]. Due to the high metabolic activity required to function, the brain utilizes approximately 60% of a body’s daily glucose intake and requires a constant supply of glucose due to its inability to store glycogen [6]. In normal brain cells, glycolytic activity in the cytosol converts glucose into pyruvate. Under normoxic conditions, pyruvate proceeds down the aerobic pathway, forming acetyl-CoA to then proceed through the tricarboxylic acid cycle (TCA). This cycle then produces flavin adenine dinucleotide (FADH2) and nicotinamide adenine dinucleotide (NADH), which then go through oxidative phosphorylation to produce a large amount of ATP. An insufficient presence of oxygen results in anaerobic glycolysis, in which pyruvate ultimately forms lactate, and this process produces significantly less ATP than the aerobic pathway (Figure 1) [7]. A defining characteristic of GBM is that even in the presence of oxygen, tumor cells still prefer anaerobic glycolysis for energy production and biomolecule synthesis, and so this characteristic of GBM is defined as the Warburg effect (Figure 1) [8]. GBMs prefer anaerobic glycolysis due to the increased production speed of ATP and other necessary macromolecules. As oxidative phosphorylation utilizes glucose more efficiently to produce a larger quantity of ATP compared to anaerobic glycolysis, tumor cells must compensate for this decreased efficiency in energy production (Figure 1). As part of the solution, tumor cells must uptake more glucose by upregulating glucose transporters (GLUTs) [9]. Furthermore, studies have shown that increased quantities of glucose in the brain are correlated with increased malignancy in GBMs. A recent study illustrated that elevated glucose levels in the brain increase the expression of chemoattractant receptors to drive GBM growth [10]. In fact, GBMs alter the expression of glycolysis-related proteins, such as p53 and STAT3, to induce cancer glycolysis. STAT3 activation induces expression of the transcription factors hypoxia inducible factor 1 alpha (HIF-1α) and c-Myc, further resulting in GBM cell proliferation [11,12]. Specifically, HIF-1α regulates cellular response in hypoxia and is responsible for activating factors necessary for pathological angiogenesis driving cell proliferation [13,14]. Additionally, HIF-1α and c-Myc are oncogene transcription factors that target the glycolytic enzymes PDK1, LDHA, HK2, and TFRC [15]. Increased expression of these enzymes promotes GBM cell proliferation, therapeutic resistance, and intracranial growth [16].

The last step of glycolysis is catalyzed by pyruvate kinase by dephosphorylating phosphoenolpyruvate (PEP) to produce ATP in addition to pyruvate, which also plays an important role in tumor proliferation. There are different isoforms of PK, which are expressed based on the different metabolic functions of different cells. Alternative splicing of one of those isoforms, PKM, results in PKM2, which is exclusively expressed by tumor cells. Due to oxidative stress, PKM2 can inhibit apoptosis by traveling to the mitochondria and phosphorylating Bcl2, propagating GBM cell viability [17,18].

Parallel to the glycolytic pathway is the PPP, which contains two phases. The oxidative branch of the PPP utilizes glucose-6-phosphate dehydrogenase (G6PD) to oxidize glucose-6-phosphate into 6-phosphogluconate, which is then further oxidized into ribose-5-phosphate. NADPH is crucial for neutralizing reactive oxygen species (ROS) to protect neurons against oxidative stress. Ribose-5-phosphate is a precursor for nucleotide biosynthesis and supports cell growth and proliferation [19,20,21]. However, GBM cells utilize both the oxidative and non-oxidative branches of PPP ribose-5-phosphate for nucleotide synthesis, allowing cancer cells to be flexible in response to varied tumor microenvironments [7]. *TP53* is a tumor suppressor gene that works to suppress glucose uptake through downregulating the transcription of GLUT1 and GLUT4 [22] but in the PPP strictly inhibits G6PD, a rate-limiting enzyme that is conversely transactivated by HIF-1α. High activation of G6PD is observed in cancer cells, and so inactivation of p53 potentially results in an increase in glucose consumption. Additionally, p53 downregulates glycolysis through transcription of *TP53*-induced glycolysis and apoptosis regulator (TIGAR) and negatively regulates the PI3K-Akt-mTOR pathway, which contributes to regulating important cellular responses such as cell growth, proliferation, and survival [23].

### 2.2. Amino-Acid Metabolism

While much research has delved into the role of metabolic reprogramming of central carbon metabolism, recent research efforts have highlighted the important role that amino acids play in supporting cell survival and growth. Classical research efforts have focused on the importance of glucose in GBM growth and survival, but amino acids, in addition to generating essential biomolecules, serve as energy sources as well [24].

Glutamine, the precursor for glutamate, is an excitatory neurotransmitter and is readily available in the brain microenvironment [25]. In certain studies, glutamine deprivation has shown slower GBM tumor growth. Glutamine is involved in the production of various non-essential amino acid (NEAA) mitochondrial substrates in GBM cells [26]. Additionally, studies have shown that glutamine stimulates mTOR complex 1 (mTORC1), a component of the PI3k-Akt-mTOR pathway, which, as defined earlier, regulates important cellular responses such as cell growth, proliferation, and survival [27,28]. Furthermore, glutamine donates nitrogen in several enzymatic reactions to produce nucleotides such as purine and pyrimidine that are crucial for GBM tumor growth and invasion. Additionally, c-Myc promotes glutamine uptake and its catabolic processes, which results in GBM displaying a glutamine dependency, as consistent production of these nucleotides through the actions of glutamine ensure that GBM maintains a high proliferative rate [7,23]. Lysine is another amino acid important to GBM growth and support, though its role is not as well described. Combining c-Myc or PD-1 inhibition with a lysine-restricted diet has been shown to inhibit tumor growth and augment the efficacy of these agents [29]. Through the process of glutaminolysis, glutamine is catabolized into α-ketoglutarate (α-KG) and glutathione (GSH), fueling the TCA, discussed in further detail later, in GBM cells and producing NADH and FADH2, which can feed into the electron transport chain. This further concludes that glutamine provides the energy necessary for GBM tumor proliferation [4].

The tumor microenvironment consists of many different types of cells, one of them being cancer-associated fibroblasts (CAFs). CAFs become active by receiving signals from the tumor and promote epithelial cell invasion. Glutamine plays an important role in the process of CAFs, and as such, glutamine depravation produces a notable effect on tumor cells. Recently, it has been demonstrated that glutamine starvation resulted in CAFs migrating towards glutamine-rich areas and tumor invasion in similar areas as well [27,30].

Additionally, several other amino acids play a key role in supporting GBM proliferation. Glutamine metabolism is a way of producing aspartate, a source of carbon atoms in purine and pyrimidine biosynthesis important for GBM tumor cells to support their rapid proliferation. Another amino acid, arginine, promotes cell adhesion in GBM cells driving tumor cell migration and invasion [31]. On the other hand, research has demonstrated that the amino acid methionine inhibits important signaling proteins such as PI3K, ultimately slowing GBM cell growth [32,33].

### 2.3. Lipid Metabolism

Lipids play a vital role in cellular processes, functioning as both an energy reserve and fundamental components of the plasma membrane. Structurally, lipids are categorized into two main types: simple and complex [34]. Simple lipids, commonly known as fats or triglycerides, are straightforward molecules predominantly used for energy storage [35]. Complex lipids, which include phospholipids and glycolipids, are integral to constructing cellular membranes and facilitating various biological processes crucial to the cell.

Understanding lipid metabolism becomes especially crucial in challenging environments, such as hypoxic conditions encountered by tumor cells. In these settings, lipid metabolism does not just support basic cellular functions but adapts in ways to meet the demands of rapid tumor growth and survival. To survive in hypoxic microenvironments, tumor cells reprogram their lipid metabolism, enhancing their biological functions and adaptability. This can be seen by the augmented use of fatty acids to fuel their growth and lipid synthesis [35]. Unlike normal cells, which primarily utilize oxidative phosphorylation, tumor cells increase lipid production by favoring, as discussed previously (Figure 1). This metabolic alteration allows tumor cells to continue thriving and growing even under low-oxygen conditions by utilizing the glycolytic intermediates, such as acetyl-CoA, which are essential for initiating de novo fatty acid synthesis [36].

Cells can increase their lipid content through internal synthesis and external uptake for metabolic activities. De novo lipogenesis (DNL) is the process through which endogenous lipids are produced, generating fatty acids from excess carbohydrates, primarily glucose, and can lead to an increase in the lipid pool within the cell [34]. This process converts these sugars into acetyl-CoA, which can be derived from citrate if ATP is present or from acetate via acetyl-CoA synthetase (ACSS) [18]. ACSS exists in different forms, most importantly as ACSS1 and ACSS2, and ACSS2 contributes to the cytosolic pool of acetyl-CoA, which serves as the substrate for fatty acid synthesis [18,37].

Once in the cytosol, acetyl-CoA is further carboxylated to malonyl-CoA by acetyl-CoA carboxylases (ACCs), enzymes that catalyze this process [38]. This reaction is a bottleneck in the process since fatty acid synthase (FASN) requires malonyl-CoA to produce saturated fatty acids [38]. DNL, which is particularly active in the adipose and liver tissue and is tightly regulated, requires this conversion of acetyl-CoA to malonyl-CoA by ACC. Meanwhile, exogenous lipids are obtained through the uptake of dietary fats or lipids absorbed from the external environment. These exogenous lipids are incorporated into cells primarily through the process of lipid absorption and transport, facilitated by specific transport molecules such as CD36, fatty acid-binding proteins (FABPs), and the fatty acid transport protein family (FATPs/SLC27) [34].

In addition to fatty acids, cholesterol is another important lipid in the brain, accounting for around a quarter of the body’s total cholesterol [39]. The blood–brain barrier (BBB) prevents the uptake of cholesterol from the blood circulation, forcing the brain to produce its own cholesterol by de novo synthesis [40]. De novo synthesis of cholesterol in the brain is generally carried out by glial cells, particularly astrocytes, rather than by neurons [40]. Therefore, the cholesterol produced by astrocytes is delivered to neurons via lipoproteins [27,41]. However, this is not the case for GBM cells. Unlike glial cells, GBM cells rely heavily on exogenously sourced cholesterol, which is crucial for their survival. The uptake of exogenous cholesterol in GBM cells is controlled by sterol regulatory element-binding proteins (SREBPs), specifically SREBP-1 [42]. SREBP-1 is a transcription factor that is highly active in GBM cells and activates the expression of cholesterol synthesis genes [43]. When cholesterol levels in GBM cells drop below a certain threshold, SREBP-1 is transported from the endoplasmic reticulum (ER) to the Golgi apparatus, where it is cleaved and activated [43]. Once activated, SREBP-1 enters the nucleus and promotes the transcription of target genes that increase cholesterol uptake and synthesis [41]. This process allows GBM cells to efficiently acquire cholesterol, supporting their metabolic needs.

To meet the high energy demand, tumor cells enhance both the uptake of lipids from the environment and their internal production through de novo lipogenesis. The synthesized lipids are used not only as energy but also directed to metabolic pathways that promote oncogenic changes such as signaling pathways and membrane structures [34,44]. Alternations in lipid metabolism not only alter the tumor’s microenvironment by promoting a landscape conducive to cancer progression but also support the proliferation of tumor cells [34].

### 2.4. Nucleotide Metabolism

Alterations in nucleotide metabolism can play a significant role in the progression of GBMs. Among the pathways that are affected by GBM reprogramming are de novo purine and pyrimidine synthesis and catabolism, nucleotide salvaging pathways, folate metabolism, mitochondrial nucleotide metabolism, and AMP-activated protein kinase (AMPK) signaling pathways. GBM cells often exhibit upregulated de novo synthesis of purine nucleotides to support their rapid proliferation [45]. Key enzymes in this pathway, such as amidophosphoribosyltransferase (PPAT) [46] and phosphoribosyl pyrophosphate synthetase (PRPS), are overexpressed or activated [47]. Like purine synthesis, GBM cells may increase the synthesis of pyrimidine nucleotides de novo to meet the demands of proliferation. Enzymes like carbamoyl-phosphate synthetase 2, aspartate transcarbamylase, and dihydroorotase (collectively known as CAD) are critical in this pathway and are also dysregulated in GBMs [48,49]. In addition to de novo synthesis, GBM cells may also upregulate nucleotide salvage pathways to efficiently recycle nucleotide bases. Enzymes involved in salvage pathways, such as hypoxanthine-guanine phosphoribosyltransferase (HGPRT) and thymidine kinase (TK), could be altered in GBMs [50]. Changes in nucleotide catabolic pathways can also contribute to nucleotide metabolism rewiring in GBMs. Altered expression of enzymes involved in purine degradation (e.g., xanthine oxidase) and pyrimidine degradation (e.g., dihydropyrimidine dehydrogenase) may occur, affecting the balance of nucleotide pools [51,52]. Folate metabolism is linked to nucleotide synthesis through the provision of one-carbon units for purine and pyrimidine biosynthesis. Dysregulation of folate metabolism, including alterations in enzymes such as dihydrofolate reductase (DHFR) and serine hydroxymethyltransferase (SHMT), can impact nucleotide metabolism in GBMs [53]. Furthermore, mitochondria play a crucial role in nucleotide metabolism, particularly in the synthesis of purine nucleotides. Dysfunctional mitochondria in GBM cells may affect nucleotide synthesis and lead to altered expression or activity of mitochondrial enzymes involved in nucleotide metabolism, such as mitochondrial dihydroorotate dehydrogenase (DHODH) [54]. Indeed, one study has shown that IDH1-mutant glioma cells are hypersensitive to DHODH-targeting compounds in using a chemical synthetic lethality screen [55]. Moreover, AMPK is a regulator of cellular energy metabolism, including nucleotide metabolism. Dysregulation of AMPK signaling in GBMs may impact nucleotide synthesis pathways by altering the activity of enzymes such as ribonucleotide reductase (RR) and phosphofructokinase (PFK) [56]. Finally, hypoxia is a common feature of the GBM microenvironment and can influence nucleotide metabolism through the activation of HIF signaling. In fact, HIF-mediated changes in gene expression, including those involved in nucleotide metabolism (e.g., ribonucleotide reductase subunit M2), may promote GBM progression, which will be elaborated on later in the review [57,58,59].

### 2.5. TCA Cycle and Oxidative Phosphorylation

**Figure 2:**
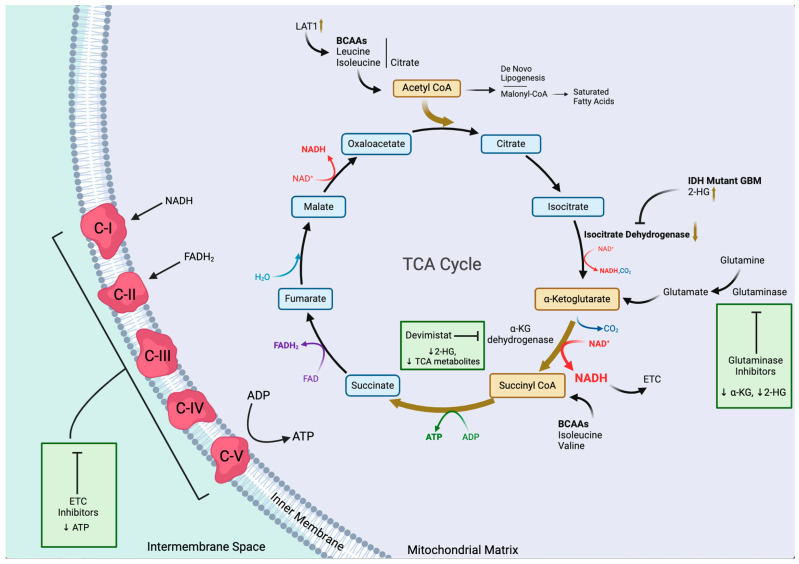
Modification of the TCA cycle by GBM tumor cells. The majority of alterations of the TCA cycle consist of modifying concentrations of 3 metabolites: acetyl-CoA, α-ketoglutarate, and succinyl-CoA. In general, branched chain amino acid (e.g., leucine, isoleucine, and valine) are catabolized at higher rates into the different intermediates of the TCA cycle illustrated in the figure. LAT1 is observed in higher levels, which is then converted into BCAAs, which then leads to an increase in levels of acetyl-CoA. Citrate is also used for conversion into acetyl-CoA, supporting the creation of saturated fatty acids. Glutamine, causing a reliance of GBM on glutaminolysis, is catabolized to α-ketoglutarate, which leads to increased production of NADH and FADH2, which feed into the ETC. Conversely, IDH is downregulated in cases of GBM with IDH mutations due to accumulation of 2-HG. Overall, increased metabolites are proposed to cause altered epigenetic regulation that increases tumor proliferation. There are several therapeutics that are under current investigation to work against the metabolic reprogramming that occurs in the TCA cycle of GBM tumor cells. ETC inhibitors work to inhibit an element of ETC (e.g., complex I, complex II, complex III, ATP synthase), thereby reducing the ATP levels in the tumor cells and eventually resulting in cell death. Devimistat is a drug that targets α-ketoglutarate dehydrogenase and is shown to reduce TCA-cycle metabolites, reduce 2-HG, and induce cell death. Glutaminase inhibitors are another form of treatment that work to reduce levels of α-ketoglutarate and 2-HG. The reduction in α-ketoglutarate is thought to be a way to reduce tumor growth. TCA, tricarboxylic acid cycle; CoA, coenzyme A; LAT1, L-type amino acid transporter 1; BCAA, branched chain amino acid; GBM, glioblastoma multiforme; NADH, nicotinamide adenine dinucleotide + hydrogen; FADH, flavin adenine dinucleotide + hydrogen; ETC, electron transport chain; IDH, isocitrate dehydrogenase; 2-HG, 2-hydroxyglutarate; ATP, adenosine triphosphate; ADP, adenosine diphosphate.

The reprogramming of the TCA cycle includes downregulation of isocitrate dehydrogenase (IDH) and reprogramming of the anaplerotic pathways [60,61,62] (Figure 2). Mutations in IDH enzymes are common in GBMs, leading to alterations in the TCA cycle, including the accumulation of the oncometabolite 2-hydroxyglutarate (2-HG), a small molecule intermediate produced from αKG [63].

Furthermore, IDH mutations, such as the ones associated with IDH1 and IDH2, have a profound impact on glioblastomas. One of the prominent effects of IDH mutations is the inhibition of αKG-dependent dioxygenases, which results in drastic genetic alteration that increases dependence on glutamate [27]. In addition, metabolic pathway changes occur in the GBM cells because of IDH mutations that further impact the TCA cycle. Hence, GBM cells rely on alternative anaplerotic pathways, which are pathways that aim to replenish TCA cycle intermediates. An example of that is the utilization of increased glutaminolysis to generate αKG [64]. Finally, GBM cells exhibit a dysfunctional oxidative phosphorylation process and defects in mitochondrial function, including impaired electron transport chain (ETC) activity and reduced mitochondrial membrane potential [65].

## 3. Drivers of Metabolic Reprogramming in GBMs

### 3.1. Hypoxia

Hypoxia, characterized by reduced oxygen levels, is a distinctive feature of the tumor microenvironment in GBMs. The depletion of oxygen occurs due to the rapid and disordered proliferation of cancer cells, surpassing the capacity of existing blood vessels to adequately deliver oxygen to the cells. Hypoxia plays a crucial role in driving the progression and aggressiveness of GBM tumors through various mechanisms, the focus of this section being metabolic reprogramming. Understanding how hypoxia promotes these aggressive characteristics is essential for developing more effective therapies.

**Figure 3:**
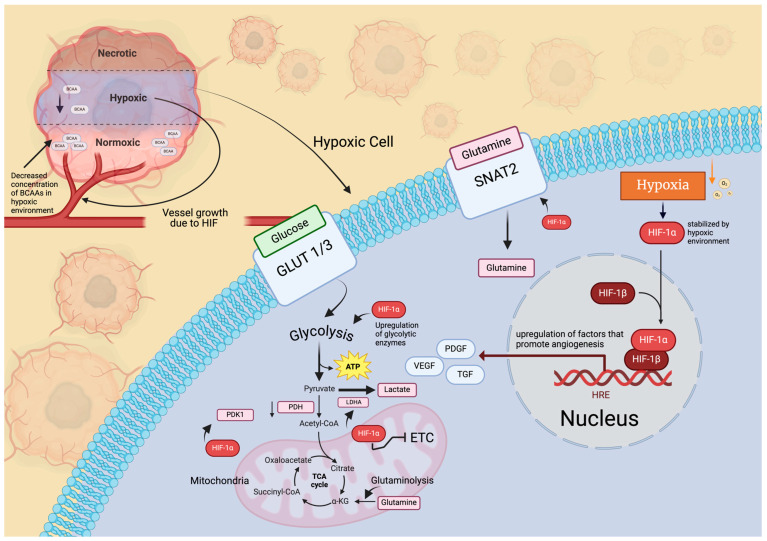
Molecular pathways altered by the onset of hypoxia in a malignant glioblastoma cell. When oxygen levels are low in a cell, HIF-1α is stabilized, allowing the transcription factor to translocate to the nucleus before proteasomal degradation. Once in the nucleus, HIF-1α assembles with HIF-1β on the HRE to then enhance transcription of HIF target genes to promote tumorigenesis by increasing cell vascularization; PDGF, VEGF, and TGF. Furthermore, the HIF-1 heterodimer has been linked to increases in the expression of glutamine transporters—enabling higher rates of glutaminolysis—and the upregulation of several glycolytic enzymes and transporters, resulting in increased lactate production. As shown, HIF-1α promotes the expression of PDK1, which decreases the activity of PDH and increases pyruvate availability for lactate production. This is coupled with the increased expression of LDHA. Lastly, while there has been no elucidation of the mechanism, there is a low concentration of BCAAs in hypoxic conditions relative to neighboring normoxic areas. HIF-1α, hypoxia inducible factor-1α; HIF-1β, hypoxia inducible factor-1β; HRE, hypoxia response element; PDGF, platelet-derived growth factor; VEGF, vascular endothelial growth factor; TGF, transforming growth factor; PDK1, pyruvate dehydrogenase kinase 1; PDH, pyruvate dehydrogenase; LDHA, lactate dehydrogenase A; BCCAs, branched chain amino acids.

HIFs are critical mediators of the cellular response to hypoxia in GBMs. Three members of the HIF family (HIF1–3) have been found in mammals, and each factor is formed by dimerization of α and β subunits (Figure 3). The heterodimer interacts with the hypoxia response element (HRE) after being translocated to the nucleus and enhances the transcription of downstream target genes. Under hypoxic circumstances, HIFs become stabilized, with HIF-1α assuming a pivotal role in governing gene expression when oxygen levels are low. HIF-1α fosters the activation of genes related to glycolysis, angiogenesis, cell survival, and metastasis, all of which are essential for the advancement of GBM. As previously stated, GBMs primarily rely on glycolysis [66], with HIF-1α serving as a key transcription factor responsible for controlling the expression of genes associated with glucose metabolism (Figure 3). Within GBM cells, HIF-1α undergoes stabilization due to the hypoxic environment, leading to increased expression of glycolytic enzymes and glucose transporters. In fact, GBM cells were found to upregulate their expression of the enzymes HX2, PFK1, ALDOA, and PGAM1 upon exposure to hypoxic conditions [67,68]. Furthermore, subsequent experiments involving the knockdown of each of the genes expressing these proteins resulted in an attenuated proliferative capacity of the cancer cells [67]. Moreover, HIF-1α induces pyruvate dehydrogenase kinase (PDK1) to phosphorylate pyruvate dehydrogenase, preventing pyruvate conversion into acetyl-CoA, thereby augmenting glucose metabolism and diverting pyruvate from the oxidative phosphorylation pathway towards lactate generation in cancer cells (Figure 3) [69]. These circumstances promote the tumor‘s metabolic shift towards the Warburg effect. In addition to affecting glucose metabolism, HIF-1 and HIF-2 are well known to reprogram cellular metabolism in other metabolic enzymes by upregulating their expression in GBM cells. In branched-chain amino acids (BCAAs), such as leucine, valine, and isoleucine metabolism, HIF-mediated reprogramming under hypoxia showed increased glutamate labeling [69]. Metabolomic studies have revealed a notable decrease in the concentration of BCAAs in hypoxic regions compared to peripheral nonhypoxic areas, while levels of glutamate remain consistent across both regions [70]. As a result, HIF-mediated reprogramming of BCAAs could be a crucial mechanism for sustaining glutamate levels in the hypoxic areas of tumors. The exact mechanism by which BCAA metabolism is reprogrammed is unknown, but the impact of this pathway under hypoxic conditions is an increase in production of branched chain aminotransferase (BCAT) and L-type amino acid transporters (LATs) (Figure 3). HIF-1 and HIF-2 are known to bind to HRE at the *BCAT-1* gene, and only HIF-1 activates mRNA transcription of the enzyme. LAT1 is catabolized into branched chain α-ketoacids (BCKAs) by BCAT, which increases the availability of metabolites of the TCA cycle, like acetyl-CoA, from which BCKAs can be derived (Figure 3) [71].

The essential result of hypoxia is an increased progression and aggressiveness of GBM tumors by the known stabilization of HIFs, especially HIF-1α. The focus of this section was the reprogramming of glucose and BCAA metabolism, both of which lead to the altered synthesis of metabolites that increase the survival and proliferation of GBM cells. It is important to mention the persistence of hypoxic regions due to the other targets of HIF-1α. The promotion of angiogenesis—the process by which new blood vessels are formed—leads to the highly vascularized trait of GBM tumors (Figure 3). But the newly formed blood vessels are often abnormal and leaky, further exacerbating hypoxia and creating a continuous cycle that sustains tumor growth [72]. These vascular structures are called glomeruloid bodies (or vascular tufts), and they lead to an increase in pseudopalisading zones around neurotic regions of the tumor. Naturally, these zones are highly hypoxic and lead to again the stabilizing of HIF-1α and continuance of the cycle previously mentioned.

Several therapeutic measures seek to target the metabolic pathways HIF alters. Inhibiting HIF-1 has been demonstrated to reduce tumor proliferation, migration, and invasion as well as to downregulate downstream genes. [73]. And in combination with radio- or chemotherapy, several drugs targeting HIF-1 α, such as apigenin and propofol, modulate its transcriptional activity [68]. However, the limited effect of these inhibitors is attributed to the lack of specificity of HIF-1α inhibitors [74]. In addition to HIF-1α, inhibiting HIF-2α can also disrupt the hypoxia pathway in glioblastoma. Recent studies have indicated that HIF-2α is overexpressed in GBM cells but not in normal tissues. However, antitumoral activity with inhibition of HIF-2α has not been shown in glioblastomas. Overcoming hypoxia-induced therapy resistance represents a major challenge in the treatment of GBMs. Due the rare efficiency demonstrated with HIF inhibitors, the current literature recommends further research into the complete blockade of HIF and not just inhibition of its downstream pathways [74]. This may offer new avenues for improving outcomes in patients with GBMs.

### 3.2. Microenvironment and Extracellular Matrix

The heterogeneity of the GBM tumor microenvironment (TME) serves as one of the main reasons for the aggressive behavior of this tumor [75]. The hallmarks of the TME architecture include an invasive edge, a hypoxic center, and a perivascular niche [76]. The TME includes noncancerous cells (stromal cells) like immune cells, endothelial cells, pericytes, and fibroblasts [77,78]. Glioblastoma stem cells (GSCs), though present in small quantities, enable the tumor to invade healthy brain tissue and resist therapeutic treatments [79]. Additionally, proteins, non-protein biomolecules (such as nitric oxide and polysaccharides), and the modified extracellular matrix (ECM) and interstitial fluid make up the tumor microenvironment (TME) and its capacity to accelerate tumor growth [80]. Metabolic reprogramming enables GBM cells to adapt to new microenvironments, aiding in invasion of surrounding brain tissue. Understanding these metabolic vulnerabilities could lead to effective therapeutic strategies [27]. This section focuses on how metabolic processes are exploited by GBM’s remodeling of components of the TME and ECM.

The Warburg effect seen in tumor cells explains lactate secretion within the TME. Lactate maintains an acidic pH that significantly promotes tumor growth, invasion and vascularization and aids in immune evasion [8]. Specifically, the acidic lactate in the extracellular space causes the recruitment of immune cells like macrophages and microglia, which then obtain a tumor-promoting immunosuppressive phenotype [8]. Stromal acidification due to lactate secretion has been shown to promote matrix metalloproteinases (MMPs), which are endopeptidases responsible for tissue remolding and degradation of the cell extracellular matrix [81]. In promoting MMPs, GBM cells can further migrate and infiltrate healthy brain cells. One example would be expression of MMP-2 and MMP-9 due to TGF-β2, which are then sustained by lactate production [77,82]. This induced expression promotes ECM remodeling and degradation, enhancing GSC invasion.

Moreover, research is ongoing regarding the role of viruses in cancer development and progression, with a focus on their ability to modify the TME, augmenting the tumor’s ability to progress and metastasize. For example, human cytomegalovirus (HCMV)-infected GBM cells alter the expression of important metabolic proteins [83]. Following infection, there is upregulation of glucose and lactate transporters and increased lactate production and export, resulting in suppressed immune response and increased angiogenesis [84]. Increased lactate can also be used as metabolic fuel potentially leading to high levels of oxidative phosphorylation. A consequence of increased oxidative phosphorylation is an observable increased expression of reactive oxygen species such as superoxide, further creating damaging conditions for cancer proliferation [85]. This finding proposes a form of virus-induced metabolic reprogramming that changes the TME to allow for increased tumor progression.

Recent studies have shown a role in the ECM for receptor and non-receptor tyrosine kinases in metabolic reprogramming by GBM cells. One study showed that proto-oncogene tyrosine-protein kinases’ (SRC kinases) activity in GBMs promotes and sustains metabolic reprogramming (and inflammation). The non-receptor kinase is a downstream intermediate of many receptor tyrosine kinases that trigger the phosphorylation of substrates involved in cell motility, angiogenesis, survival, adhesion, and proliferation [86]. Regarding the ECM, SRC kinases affect the distribution of proteins that form focal adhesions and regulate integrin signaling and ECM protein expression, resulting in reduced cell–cell interaction and cell–ECM adhesion, thereby supporting changes in the TME.

Another recent study showed that a soft, brain-like matrix causes GBM cells to shift to a glycolysis-dominant metabolic state through CD44 and integrin receptors, an overall state which supports invasive behavior [87]. However, the study was conducted in normoxic conditions, which are unlike the true hypoxic GBM TME. Nevertheless, studies such as this contribute to an increased understanding of the relationship among ECM mechanics, cell metabolism, and tumor progression.

Overall, the interaction between GBM tumor cells and TME nontumor cells happens through reciprocal exploitation of metabolic changes initiated by cancerous cells—metabolic rewiring—to promote tumor progression and resistance [77]. Murine slices of decellularized brain ECM (dBECM) shown to mimic microenvironment dynamics during glioblastoma infiltration showed reprogrammed glucose metabolism [88]. This highlights the implication of the native brain ECM in driving metabolic reprogramming and subsequent treatment-resistant phenotypes.

### 3.3. Immune System

Immunotherapy in GBMs has shown limited benefit from current approaches due to complexities of the tumor immune microenvironment (TIME). Tumor cells adapt to demands for energy and cellular building blocks to sustain differentiation, rapid proliferation, and survival [89]. Within the TIME, cancer cells continuously interact and exchange signals with neighboring stromal and immune cells through various secreted factors, such as cytokines and chemokines. Current research highlights that GBM cells in the TIME can suppress anti-tumor immune cells. This immunosuppression is what allows for continued growth of the tumor.

Microglia, derived from early hematopoietic stem cells in the yolk sac, play a nuanced role in modulating GBM growth by driving immunosuppression [90]. Some studies have shown that the expression of oxidative stress-related genes is augmented in microglia obtained from tumors as comparted to those present in normal brain parenchyma. These findings suggest that oxidative stress reprograms microglia into an activated state, which then enhances the growth and aggressiveness of GBM cells. Furthermore, the level of nuclear receptor (NR) 4A2 in microglia is higher in GBMs than in lower-grade gliomas, indicating that oxidative stress induces NR4A2 activation in microglia [90]. NR4A2 is functionally important to the increased expression of genes related to lipid metabolism and decreased expression of genes involved in the MHC-1 complex and antigen presentation [90]. Therefore, the induced phenotypic changes in microglia lead to altered metabolic change in fat production necessary for the growth of GBM cells and increase immunosuppression by preventing the detection of antigen presence to functioning immune cells, thus allowing GBM to avoid immune cell surveillance. In addition, glioma-associated microglia/macrophages (GAMMs) are recruited into the TIME and participate in the creation of an immunosuppressive environment [91]. While this is documented, there is compelling evidence that illustrates the contrary. A study examining the role of microglia in breast cancer brain metastasis found that microglia support tumor suppression in the TIME [92]. Another study investigating Prolyl 4-hydroxylase subunit a1 (P4HA1) knockdown concluded an increased polarization of microglia to the M1 phenotype, a morphology known to induce cytotoxicity and inflammation. As a result, GBM cells had a lower proliferative capacity and were less likely to invade healthy tissue [93].

In addition to GAMMs, myeloid-derived suppressor cells (MDSCs) with significant immune-suppressing action (e.g., neutrophils and monocytes) [94] are also greatly recruited into GBM tumors. They are said to govern two major metabolic pathways in the GBM: (1) fatty acid oxidation using PPARγ and AMPK and (2) glycolysis using HIFα and mTOR [89]. GBM cells drive the induction of MDSCs through the release of extracellular vesicles [95]. In IDH-mutant GBMs, infiltrating myeloid cells use 2-hydroxyglutarate and tryptophan degradation to generate an immunosuppressive TIME [96].

In the TIME, reactive astrocytes supply the metabolic requirements of tumor cells [97]. Most microglial cells in benign or regressing tumors have pro-inflammatory activity that promote tumor lysis through expression of inducible nitric oxide synthase (iNOS). But astrocyte depletion regulates tumor-associated macrophage (TAM) cytotoxic potential and can modulate nitric oxide metabolism in the TIME. In short, astrocytes in GBM tumors can play an important role in reprogramming other immune cells in the glioma microenvironment. Current research shows that TAMs in solid tumors do not play an antineoplastic role but rather help the cancer cells in creating an immunosuppressive environment [98]. This plasticity that TAMs exhibit makes them an appealing target for immunotherapy.

Other immune-relevant factors that drive metabolic reprogramming in GBMs include CD97, tryptophan, adenosine, hexokinase 3, d-2-hydroxyglutarate (d-2HG), Pit-Oct-Unc (POU) class Homeobox 2, CD147, and ACOD1 gene. Herein, CD97 is linked to the activation of the MAPK/Erk and PI3K/Akt pathways in GBMs [99]. Tumors exhibiting high levels of CD97 expression were connected to alterations in the TIME, such as an increase in resting NK cells, naïve macrophages, and regulatory T cells. Tryptophan is an amino acid that is converted to kynurenine within tumor cells. This is carried out by the rate-limiting enzymes indoleamine 2,3-dioxygenase (IDO) and tryptophan 2,3-dioxygenase (TDO) and contributes to further suppressing immune cells. Adenosine is an anti-inflammatory molecule that plays a role in restoring tissue homeostasis by modulating the innate and adaptive immune responses. While adenosine is normally present at low levels outside cells, necrotic tumors produce high levels of it, which are then metabolized by cell surface ectoenzymes. This process contributes to an immune-suppressive state [100]. In a recent study, immune cell infiltration into the TIME was increased by hexokinase 3 (HK3) [101]. Samples of tumor tissue with higher HK3 showed increased levels of infiltration of different kinds of memory CD4+ T cells, neutrophils, and M2 macrophages. It was also noted that HK3 expression increased as tumor grade increased in the mesenchymal subtype of GBMs compared to others. Moreover, d-2HG is a molecule produced by mutated isocitrate dehydrogenase (IDH), while lactate dehydrogenase (LDH) has been identified as a target of d-2HG. Hence, the effect of this relationship is the inhibition of LDH activity in CD8+ T cells. Exhibited in a study of patients with mutations in IDH1 and IDH2 were reduced levels of CD8+ T cell cytotoxicity and weakened interferon-γ signaling, both of which contribute to an immunosuppressive TME [102]. The POU class Homeobox 2 (POU2F2) controls genes related to B cell proliferation and differentiation. Elevated expression levels of POU2F2 are significantly associated with a poor prognosis in GBM patients. This suggests that POU2F2 induces a metabolic change towards aerobic glycolysis, thereby promoting the progression of GBM [103]. The CD147 immunoglobulin activates HIF-1/2α, which leads to changes in glycolysis and amino acid metabolism [104]. Lastly, the ACOD1 gene is an important gene involved in reprogramming macrophages towards an anti-inflammatory phenotype. In a recent study, decreased antigen-presenting cell signatures on TAMs are retained under ACOD1 deficiency, indicating that ACOD1 absence turns macrophages to a more reactive and immunogenic phenotype [105].

Overall, understanding the biochemical dialogue among tumor and immune cells in the TIME is crucial for developing effective therapies for GBMs. Many therapies have been introduced to target immunosuppression such as control of TAMs [106], inhibition of CD97 [99], and disruption of GBM EV–monocyte interactions [95]. However, these approaches have not provided stronger median survival rates than that of standard treatment with chemoradiotherapy.

### 3.4. Stem Cells and Nanotubes

Stem cells are highly involved in the metabolic reprogramming observed in GBMs. Cancer stem cells (CSCs) are pluripotent and neoplastic cells which have the capacity to produce new stem cells of different kinds that can replenish the cell population in tumors [107]. CSCs demonstrate a highly dynamic plasticity, at both the metabolic and transcriptional levels, that supports this heterogeneity. The heterogeneity of CSCs is also responsible for altering many mechanisms within the CSCs and the tumors themselves, such as drug responsiveness and metabolic activity. CSCs generally have a slow but steady proliferation. This torpid mitotic activity can permit a CSC’s evasion of treatments that target cells that are actively dividing [107]. In the context of GBMs, there is a population of cells that shares properties with CSCs and is referred to as glioma/glioblastoma stem cells (GSCs). There are two types of GSCs that are recognized—proneural (PN) and mesenchymal (MES) GSCs [107]. They are both found in GBMs, and they proliferate at different rates as well as respond to treatment differently. MES GSCs have demonstrated a higher proliferation rate, a more aggressive rate of invasion, and more resistance to radiation than PN GSCs. Though PN GSCs are known to be less hostile, they possess the ability to convert to MES GSCs. It was found that following treatment, these PN GSCs usually drive tumor relapse and present with MES-like markers. These relapsed GBM cells were also found to be more resistant to chemotherapeutics. The exact mechanism by which this phenomenon occurs remains unknown, but it does demonstrate the GSC’s ability to be reprogrammed in order to optimize survival [108]. In the context of GSCs, there is the niche concept which describes the location of the GSCs within the tumor. There are three primary niches: perivascular, invasive, and hypoxic. Each niche has a mode of interaction with the GSCs that is relevant to metabolic reprogramming in GBMs. As an example, invasive niches contain GSCs that grow perivascularly along the capillaries, in between the endothelium and the astrocytic end-feet [109]. These GSCs are nestin-positive and ultimately result in the displacement of the astrocytic end-feet and pericytes of the neurovascular unit. This disruption of the neurovasculature promotes angiogenesis for the tumor. In the hypoxic niche, GSCs are induced through the mechanisms of HIFs. In the perivascular niche, there is considerable interaction between the GSCs and the endothelium [109]. These interactions are quite significant. For one, the endothelium expresses DLL4 and JAG1, which are signaling proteins. Notch 1 and Notch 2 receptors are expressed by GSCs, and they induce a response to the signaling proteins released by the endothelium. This activation of Notch leads to the activation of the *HES1* and *HEY1* genes. The activation of these genes is correlated with promoting the proliferation of GSCs [110,111,112]. WNT is another signaling pathway which has shown involvement in the metabolism of GBMs. The mutation of *FAT1* results in an improperly activated WNT pathway, which results in tumor growth. Another pathway that is relevant to GSCs and their activity is the Sonic hedgehog (SHH) pathway. The SHH pathway results in the activation of *GLI1* and *GLI2* as downstream effectors. These proteins bind to the Nanog promoter, which ultimately upregulates the production of certain stemness factors. Usually, p53 exerts an inhibitory action on Nanog; however, in the context of GBMs, the function of p53 is disrupted and often lost. This interference with the function of p53 further contributes to the activation of SHH and Nanog, which results in an augmented expression of stemness factors [107]. Generally, the TME and the molecular makeup of the GSCs are the main drivers of their metabolism. In the perivascular niche, the GSCs are generally of the PN variety and have a predominant glycolytic metabolism. In these PN cells, the glucose can either be converted to lactate or go through oxidative phosphorylation. Notably, these cells also show an elevated expression of glutamine synthase (GS). This enzyme allows for the direct synthesis and secretion of glutamine. In the hypoxic niche, the GSCs are usually of the MES variety and have a metabolism suited for the challenging environment. The metabolism is fueled by substances like lactate, ketone bodies, and amino acids that are released into the TME by other cells. These substrates are then used to supply a docked TCA cycle in order to generate energy and other products such as nucleic acids or lipids [113]. Notably, the difference between the two metabolic profiles is that in the hypoxic niche, there is no expression of GS. Hypoxia favors the self-renewal of the GSCs. These GSCs are known to promote the conversion of microglia/macrophages to the M2 subtype. These M2 type immune cells are more aggressive and damage the parenchyma. They also promote the survival and growth of the tumor through their release of several cytokines and chemokines [113]. Additionally, recent studies have shown that M2 macrophages induce the production of the PDGFRβ in GBM cells and stimulate their migration [114,115]. GSCs are also known to differentiate into pericytes whenever angiogenesis is occurring in the tumor. This ultimately leads to the support and the growth of the tumor. Increased pericytes along with a disrupted BBB is a marker for active angiogenesis in the tumor [109]. Additionally, over-activation of the PPP can be observed in GSCs. The PPP is more active in cancer cells that are proliferating under normal oxygen conditions than those cells that are located in a hypoxic environment. This pattern of activation correlated with oxygen levels is seen inversely for the glycolysis pathway. Under normoxic conditions, the activity of the glycolysis pathway is lower than in hypoxic conditions (Figure 1). This is a reciprocal metabolic relationship whose effect is described by the phrase “go or grow.” This relationship allows for the switch between the PPP and glycolysis in these GSCs that is dependent on the environment. The dominance of either pathway at any given moment is determined by the condition in which the tumor finds itself. A lack of oxygen results in the migration of GSCs and in the reduction of proliferation, while a properly oxygenated environment results in the reduction of GSCs’ mobility and an increase in proliferation. It is thought that a dominance of the PPP is correlated with a highly proliferative state in the GSCs, while the dominance of the glycolytic pathway is correlated with a highly migratory state in GSCs. The idea is that the conditions determine whether the cells will invade/migrate (“go”) or proliferate (“grow”) [107,113].

In a related context, long, thin, non-adherent, open-ended protrusions in the cell membrane called tunneling nanotubes (TNTs) have also been greatly implicated in metabolic reprogramming in GBMs. They serve as a communication method within tumor cells as well as between tumor cells and stromal cells. TNTs form large networks with high connectivity [116]. Overall, TNTs have been shown to exchange an array of molecules and organelles, such as mitochondria, death signals, and miRNAs and are even implicated in the maintenance of the niches that were discussed previously [117]. Their formation is notably accelerated in response to cellular stress. This includes the presence of reactive oxygen species (ROS) and ROS-inducing chemotherapies. This is especially problematic because TNTs introduce methods by which certain cells can rescue each other from therapeutic-induced death [118]. Communication is essential to the growth and spread of tumors, and TNTs seem to facilitate the transmission of information and interaction between the cells composing the tumor. The most relevant cargo exchanged through the TNTs is mitochondria [116]. Several studies have demonstrated that the transfer of mitochondria is prevalent in GBMs and is relevant to the development of chemoresistance and cell proliferation, migration, and metastasis [119]. A study performed with human mesenchymal stem cells (MSCs) and cancer cell lines demonstrated an exchange of mitochondria between the MSCs and the cancer cells. The metabolic changes that ensued included increased oxidative phosphorylation and ATP production. These metabolic changes resulted in enhanced proliferation and invasion in the cancer cells. A subsequent study indicated that mitochondrial transfer via nanotubes can occur in GBM cells specifically [120]. Mitochondrial transfer causes a number of metabolic changes in the recipient cells. To start with, TNTs form between GBM cells and stromal cells. This demonstrates a mechanism of recruitment of normal cells into the tumor, which requires a massive amount of overall metabolic changes [119,120]. There have also been studies showing that TNTs are formed by GBM cells to non-cancerous astrocytes. These TNTs allow the transfer of mitochondria to the astrocytes, which leads to the astrocytes being recruited into the tumor. TNTs and mitochondrial transfer have also shown a correlation with relapse. This mechanism is thought to involve MSCs and GSCs. It has been observed that MSCs are recruited to GBMs and that they are an origin of the transferred mitochondria. The transferred MSC mitochondria are a major driver of metabolic reprogramming within the tumor cells [120]. Some notable changes include a shift from the primary metabolic use of glucose to glutamine, a rearrangement of the TCA cycle from glutaminolysis to reductive carboxylation, a rise in orotate turnover, and enhanced pyrimidine and purine synthesis. The change seen in the TCA cycle due to the transferred mitochondria also includes a general increase in production of metabolites involved in the TCA cycle. This increased production of TCA metabolites results in an immoderate epigenetic regulation of cancer cell genes by interfering with the activity of DNA and histone demethylases. The disrupted demethylases could potentially be implicated in removing methyl groups from tumor-suppressor genes; hence, their inhibition can drive tumor growth. As for the effects seen on energy metabolism specifically, there is an increased oxygen consumption rate, which also indicates an increased level of oxidative phosphorylation and ATP production [119,120,121]. These enhanced metabolic pathways indicate overall enhancement in the metabolic activity of the GSCs due to the transferred mitochondria. A result of this enhancement is the promotion of tumor progression as well as the development of therapeutic resistance [120]. The exact cellular mechanisms by which the transferred MSC mitochondria accomplish these changes in the tumor cell metabolism remain largely unknown. Additionally, it has been shown that MSC presence in GBMs rises following radiotherapy and is inversely correlated with patient survival rates. In a similar fashion, a relationship between MSC mitochondria and TMZ resistance was demonstrated through the inhibition of the production of orotate, which is one of the changes in metabolism due to transfer of the MSCs mitochondria into the GBM cell [120]. This inhibition causes the regain of chemotherapeutic sensitivity in the GBM with transferred mitochondria, which indicates that the effects of these transplanted organelles are involved in the chemoresistance that develops in GBMs. These results ultimately highlight a mechanism of the metabolic reprogramming in GBMs that results in the resistance to chemotherapeutic drugs and may indicate how TMZ resistance in GSCs depends on a higher orotate turnover [119,120,122].

### 3.5. Effects of Therapeutic Drugs

Metabolic reprogramming in GBMs can be induced by therapeutic drugs. This ability in tumors to adapt their metabolism in response to therapeutics can be referred to as post-therapy intratumoral heterogeneity, and it represents the genetic and metabolic alterations that occur in tumors after treatment [123]. Currently, the standard treatment for a GBM includes temozolomide (TMZ). Although it is an effective alkylating agent, it typically does not eradicate all GBM cells. This indicates that there are a portion of cells that survive the alkylating actions of TMZ, primarily the previously discussed GSCs. Their low proliferative activity is protective against the alkylating damage induced by TMZ [124]. In this context, CD133 is a widely accepted marker of GSCs, and when CD133+ GSCs and CD133-GBM cells are compared in terms of chemoresistance, it has been shown that CD133+ GSCs demonstrate more chemoresistance than CD133-GBM cells. GSCs are more capable of avoiding apoptosis and activating their DNA repair mechanisms in response to an alkylating agent like TMZ [123]. It is suggested that polycomb group protein Bmi-1 (PGPB1) might be involved in this resistance. PGPB1 has a critical role in maintaining GSCs homeostasis in response to the presence of ROS. The activated PGPB1 is known to stimulate DNA damage repair mechanisms that could combat the actions of TMZ [125,126]. Another example is PDK1, which inactivates pyruvate dehydrogenase (PDH). Inactivating PDH eliminates the production of acetyl-CoA which in turn inhibits the TCA cycle. This dysregulated TCA cycle is characteristic of a GBM [127]. Another effect of TMZ administration is the activation of the WNT pathway in tumor cells. One of the ways that the WNT pathway contributes to chemoresistance is through the activation of O6-methylguanine-DNA methyltransferase (MGMT). MGMT is a protein that mediates DNA repair. In the presence of DNA alkylation by TMZ, MGMT preserves the genome through the removal of alkyl groups [127,128]. This dysregulation of MGMT may have downstream effects on metabolism that contribute to the actions of the GBM. For instance, MGMT has been demonstrated to be relevant in lipid metabolism. An assay demonstrated a marked difference in the lipid metabolism between MGMT methylated and unmethylated GBMs. The study ultimately suggests that the actions of MGMT have effects on metabolism and that the methylation state of MGMT can be used as a biomarker for lipid metabolism [129]. The administration of TMZ also results in the activation of the transcription factor NRF2. Under normal conditions, NRF2 is inhibited through degradation. The inhibition of NRF2 is lifted by the TMZ-induced accumulation of ROS, and it is subsequently able to promote the transcription of antioxidant genes. The release of NRF2 has been shown to be involved in autophagy, which is a method by which GBM cells escape cell death, as well as mesenchymal transition and invasion in GBMs [130].

HDAC1 and HDAC2 are involved in the regulation of glycolysis through the activation of c-Myc, which in turn controls glycolysis-related enzymes and transporters. Romidepsin and Panobinostat, which act as HDAC1/2 inhibitors, have been shown to lead to the inhibition of the c-Myc super-enhancer in GBM cells and therefore cause a loss of glycolytic activity. This inhibition of HDAC1/2 also elicits an overall reduction in glycolytic metabolites [124,127]. In a parallel fashion, drugs that target Aurora kinases have been an area of developing promise. A study that investigated the actions of an Aurora kinase inhibitor, TAK901, revealed that the drug reduced proliferation and migration and induced cell cycle arrest. Interestingly, the drug functions by altering SREBP1-mediated lipid metabolism [61]. In fact, AURKA is implicated in the regulation of glycolysis, and interference with its action results in a reduction in GBM growth. In this context, studies have also indicated that alisertib, an AURKA inhibitor, is more effective in conditions that have higher glycolytic activity. This has also been supported by studies in which it was observed that GBM cells that have more oxidative/aerobic metabolism appear to have the ability to evade the AURKA inhibitor [131].

### 3.6. Epigenetic Factors

Epigenetic factors are also involved in metabolic reprogramming in GBMs. Isocitrate dehydrogenase gene (*IDH*) mutation is a very common divergence in GBMs. A mutant IDH enzyme displays an action that converts α-KG to 2-hydroxyglutarate (2-HG). 2-HG has an inhibitory action on chromatin-modifying enzymes called 2-oxoglutarate-dependent dioxygenases. 2-HG is also known to induce a distinct epigenetic phenotype in GBM tumors called the CpG island methylator phenotype (CIMP). This state is characterized by massive amounts of hypermethylation of the promoter CpG island sites. This epigenetic change inactivates numerous tumor-suppressing genes that further support the development of GBM cells. Mutant IDH has also been implicated in the induction of histone hypermethylation. Specifically, (R)-2HG, an enantiomer of 2-HG, has recently been shown to inhibit KDM5 histone lysine demethylases. This inhibition of KDM5 is shown to play a role in the transformation of gliomas that have IDH mutations [132]. The development of IDH-mutant gliomas has also been demonstrated to be furthered by the production of the glutamate dehydrogenase 2 (GLUD2) protein. GLUD2 is involved in catalyzing the conversion of glutamate to α-KG and had been found to be specific to IDH-mutant cells [133]. In GBMs, there are other epigenetic changes that are often observed, and they include a distortion of chromosomal topology and a promotion of malignant progression of the GBM. For instance, it has been shown that the activation of the WNT pathway is influenced by epigenetic mechanisms. Other epigenetic regulations including DNA and histone modifications (methylation, alkylation, phosphorylation, etc.), nucleosome rearrangements, and non-coding RNA-mediated are seen in GBMs.

Methylation of glycolytic genes is a type of epigenetic modification that is common in the context of GBMs [134]. For example, PKM2 is an enzyme that can influence the fate of pyruvate in glycolysis. The expression of PKM2 is correlated with a decreased intensity of methylation of the *PKM* gene in GBMs [135]. DNA methylation can also regulate the function of mitochondria and oxidative phosphorylation, as seen in the example of 5-azacytidine and vitamin C. Herein, 5-azacytidine and vitamin C are DNA demethylation agents, and they impair the mitochondrial DNA levels of GBM cells, which ultimately ends up reducing the ATP supply. This reduced ATP concentration is in line with the conditions in which GBM thrives [136]. Notably, histone methylation affects cell metabolism of GBM through many enzymes. Histone methyltransferases G9a and GLP exert their actions on *HIF-1α* and ultimately suppress the transcriptional activity of HIF-1. This interfered activity of HIF-1 ultimately hinders the expression of its target genes, which include *PTGS1* and *NDNF*. The work of these enzymes also results in the inhibition of glycolysis, as HIF-1 is a transcriptional factor that is responsible for activating certain glycolytic genes [134]. EZH2 is another histone methyltransferase that binds to the EAF2 promoter and leads to the upregulation of H3K27me3 levels. This dysregulation promotes the transcription of EAF2, which results in the promotion of HIF-1α signaling. The HIF1α signaling ultimately brings about a metabolic tipping away from mitochondrial respiration in favor of glycolysis in GBM [134,137]. In a parallel manner, histone acetyltransferases (HATs) attach acetyl groups to lysine residues on histones. This acetylation opens up the chromatin in order to allow for transcription. Alternatively, histone deacetylases (HDACs) undo acetylation, which closes off the chromatin which favors transcriptional silencing [134,138]. These acetylation mechanisms also play a role in the regulation of cell metabolism in GBMs. HATs have been shown to have a correlation with the support of the proliferation of GBMs. HDACs, however, seem more notably implicated in the regulation of GBM metabolism. For example, the inactivation of certain HDACs can lead to *FoxO1* and *FoxO3* acetylation. The acetylation of *FoxO1* and *FoxO3* induces an increased expression of c-Myc, which in turn increases glycolytic metabolism in GBM.

## 4. Targeting Metabolic Pathways and Reprogramming

### 4.1. Glycolysis (Warburg Effect) and the PPP

A majority of research regarding the targeting of metabolic pathways in GBMs has been directed towards glycolysis. Most treatments targeting the glycolytic pathway that have reached clinical trials have been dietary interventions, such as the ketogenic diet (KD), which aims to limit the carbohydrate intake of patients to reduce the amount of glucose available for cancer glycolysis (Table 1) [139,140]. Pre-clinical studies regarding this diet are ongoing, but results have demonstrated promising results, with one study demonstrating that KD in combination with radiotherapy and TMZ had a greater efficacy in inhibiting tumor growth and increasing the survival of the mouse models. Furthermore, KD as a treatment is easy to implement and requires lifestyle changes from the patient instead of financially costly treatments [141].

Studies have been conducted to determine the efficacy of targeting certain proteins that play important roles in GBM aerobic glycolysis and the PPP. A recent study determined the role of *Praja2*, a protein-encoding gene, in supporting tumor glycolysis. Praja2 regulates the presence of KSR2 within a cell, which in turn regulates AMPK. Therefore, Praja2 expression results in a downregulation of AMPK, which is a key regulator of cellular metabolism and a stimulator of oxidative phosphorylation. The effect of glucose deprivation induces AMPK activation, inhibiting tumor glycolysis and suppressing GBM growth. Silencing *Praja2* achieves a similar effect in which AMPK activation increases, leading to suppression of cancer glycolysis. Additionally silencing *Praja2* enhances oxidative phosphorylation (Table 1) [142]. Researchers found that delivering inhibitory RNA molecules to the brain to target *Praja2* increased the survival rate of the mice. However, researchers modified the delivery method and delivered the RNAi via self-assembling nanoparticles (SANPs) instead of lipid nanoparticles due to their toxicity in the peripheral environment of the mice [136]. Additionally, SANPs are easier to scale, accelerating the number of potential treatments that can be implemented clinically and also allowing for a potential greater availability of this treatment. Utilizing SANPs in conjunction with chemotherapy drugs like TMZ greatly increases the susceptibility of tumor cells to the chemotherapy drugs [143].

Another method of suppressing tumor glycolysis is by targeting glucose uptake transporters. Several molecules and drugs have been discovered to inhibit the activity of different glucose transporters. A study showed the efficacy of fasentin in inhibiting glucose uptake by directly binding to GLUT1, inhibiting its activity [144,145]. Other GLUT1 inhibitors include ritonavir, which has demonstrated a reduction in glucose consumption in vitro [27]. A newly discovered potent glucose uptake inhibitor named piperazin has led to the creation of a piperazin derivative called glutor. Glutor targets GLUT1, GLUT 2, and GLUT3, further inhibiting cancer glycolysis (Table 1) [146]. Co-treatment of glutor and glutaminase inhibitor CB-839 has shown greater potency at inhibiting tumor cell growth in the presence of high glucose and glutamine levels. Thus, there is a potential therapeutic application in which the efficacy of other cancer treatments such as TMZ are increased because of the glutor and glutaminase inhibitor combination despite the nutrient-rich tumor microenvironment. The challenge here, though, is finding glucose uptake inhibitors that do not disrupt activity in normal tissue, which could lead to potential clinical side effects [139]. Targeting certain proteins and pathways involved in cancer glycolysis has also yielded beneficial results. Herein, 2-deoxyglucose (2-DG) is a glucose analogue that acts as a competitive inhibitor of hexokinase, which is the enzyme that phosphorylates glucose to initiate the glycolytic pathway. Therefore, the actions of phosphorylated 2-DG can inhibit glycolysis and suppresses GBM growth [147]. Importantly, combining 2-DG with radiation has resulted in a greater radiosensitivity of GSCs through inducing endoplasmic reticulum stress (Table 1) [148]. Researchers have also recently identified and defined the role of Gboxin, a recently discovered small molecule, as an inhibitor of ATP synthase activity and ultimately of oxidative phosphorylation. Further research has also shown that GBM allografts and patient-derived xenografts display significant sensitivity to Gboxin, further solidifying the compound as a promising therapeutic agent in GBM treatment (Table 1) [149].

**Table 1:** Summary of metabolic pathway components targeted in glioblastoma treatment.

Section	Targets	Treatments	References
Glycolysis (Warburg effect) and pentose phosphate pathway	Glycolytic pathway (carbohydrate intake)	Dietary interventions (ketogenic diet)	[139,140]
	Praja2	Praja2 silencers/inhibitors	[142]
	Glucose uptake transporters (GLUTs)	Fasentin (GLUT1), ritonavir (GLUT1), idinivar (GLUT1), piperazin, glutor (GLUT1, GLUT2, GLUT3)	[27,145,146]
	2-deoxyglucose (2-DG)	Phosphorylation of 2-DG	[147,148]
	Gboxin	Currently under investigation	[149]
	PIKE-A	PIKE-A knockdown	[150]
	HIF-1α	HIF-1α inhibitors	[13,15,151]
	PI3-Akt-mTOR pathway	Currently under investigation	
	Both the oxidative and non-oxidative branches of the PPP	DHEA (G6PD inhibitor), genistein, and imatinib mesylate	[22,23,152]
Amino-acid metabolism	Glutaminase (GLS)	GLS inhibitors (e.g., BPTES, GLS1 inhibitor)	[153]
	GLUD1	GLUD1 inhibitors (e.g., R162) combined with docetaxel	[154,155]
	mTOR	mTOR inhibitors (e.g., AZD8055)	[156]
	Relevant signaling proteins (e.g., PI3)	Methionine	[31]
	Glutamine	L-asparaginase, further research required to mitigate drug toxicity	[157,158]
	Branched-chain amino acids, BCAT1	BCAT1 inhibitors	[26,159]
Lipid metabolism	ATP-dependent citrate lyase (ACLY)	ACLY inhibitors (e.g., hydroxycitrate, SB-204990, bempedoic acid)	[160]
	Cholesterol synthesis	Bempedocic acid	[161]
	Acetyl-CoA carboxylase (ACC)	Currently under investigation	[162]
	Fatty acid synthase (FASN)	FASN inhibitors (e.g., cerulenin, C75, orlistat, C93, TVB-2640 in combination with bevacizumab) combined with chemotherapy and radiation	[163,164]
Nucleotide metabolism	Amidophosphoribosyltransferase (PPAT) (de novo purine synthesis)	PRPP analogs, molecules that target the function of PPAT	[161]
	Purine synthesis	Compounds that inhibit phosphoribosyl pyrophosphate synthetase	[161]
	Carbamoyl phosphate synthetase 2, aspartate transcarbamylase, and dihydroorotase (de novo pyrimidine synthesis)	CAD inhibitors	[162]
	Dihydroorotate dehydrogenase (pyrimidine synthesis)	DHODH inhibitors	[55]
	Hypoxanthine-guanine phosphoribosyltransferase (HGPRT)	Hypoxanthine-guanine phosphoribosyltransferase (HGPRT) inhibitors	[163,164]
	Tyrosine kinase (TK)	TK inhibitors	[163,164]
	Xanthine oxidase (purine and pyrimidine catabolism)	Xanthine oxidase inhibitors	
	Dihydropyrimidine dehydrogenase	Dihydropyrimidine dehydrogenase (DPD) inhibitors	[165]
TCA cycle and oxidative phosphorylation	Glutaminase	Glutaminase inhibitors (e.g., CB-839)	[166]
	α-KG	Currently under investigation	[64]
	OXPHOS/ETC	ETC inhibitors that target components of mitochondrial ETC: such as complex I (e.g., metformin), complex II (e.g., sorafenib), and complex III (e.g., antimycin A)	[142,167,168,169,170]
	Mitochondrial metabolism	Mitochondrial metabolism inhibitors that target components of mitochondrial function: such as inhibitors of ATP synthase (e.g., oligomycin) or mitochondrial pyruvate carrier (e.g., UK-5099), AMPK inhibitors	[171,172,173,174]
	Pyruvate dehydrogenase and α-KG dehydrogenase	Devimistat	[121,175]
	TCA cycle and cellular respiration	Imiprodones	[121,176]

As previously elaborated, HIF-1α is also a potential therapeutic target. Indeed, the HIF-1α inhibitor acriflavine has been shown to prolong the survival of orthotopic models of GBM when administered intracranially. However, it is the combination of therapies, specifically ACF, TMZ, and radiation therapy, which produces the greatest effect on tumor cell apoptosis. Additionally, a more gradual release of ACF onto the tumor bed produces the best results while limiting local toxicity, and so more research can be performed to formulate more efficient methods of release (Table 1) [73,165]. Furthermore, p53 is responsible for down-regulating glycolysis through TIGAR transcription and negatively regulating the PI3-Akt-mTOR pathway. Thus, the PI3-Akt-mTOR pathway is a tractable target for suppressing GBM growth, proliferation, and survival (Table 1). Finally, both the oxidative and non-oxidative branches of the PPP are involved in GBM tumor proliferation and survival. Hence, targeting these pathways using drugs like DHEA, which is a G6PD inhibitor, or other non-specific inhibitors such as genistein or imatinib mesylate might prove efficacious in reversing PPP reprogramming in GBMs. Additionally, utilization of drugs like DHEA and OT may increase the efficacy of chemotherapeutic drugs. Furthermore, as DHEA and OT primary interfere with cell duplication via synchronization of tumor cells in their G1 phase, these drugs may potentially be used to improve the efficiency of G1-specific therapeutic drugs (Table 1) [22,23,152].

### 4.2. Amino-Acid Metabolism

Recent research has defined several potential targets for rewired pathways of amino acid metabolism in GBMs. The primary amino acid involved in most targeting strategies is glutamine. As glutamine is crucial for nucleotide synthesis that allows GBM cells to sustain rapid proliferative activity, multiple targets exist within this pathway. Glutaminase (GLS) breaks down glutamine into glutamate and has been considered to be a possible target due to its elevated expression in cancer cells. Bis-2-(5-phenyl acetamido-1,2,4-thiadiazol-2-yl) ethyl (BPTES) is an oral GLS1 inhibitor that has demonstrated therapeutic effects against human lymphoma B cells (Table 1) [153]. Earlier studies have demonstrated that inhibition of GLUD1 (a glutamine dehydrogenase) inhibits cancer cell proliferation. Specifically, research conducted on R126, which is an inhibitor of GLUD1, showed reduced proliferation of glioma cells in xenograft mouse models and in vitro studies. Additionally, the study demonstrated that a combination of R162 and docetaxel inhibited cancer cell growth both in mouse models and in vitro, suggesting therapeutic potential in suppressing GBM proliferation (Table 1) [154,155]. In a similar fashion, targeting mTOR, which is involved in glutamine metabolism, has led to GBM cell proliferation suppression (Table 1) [27]. AZD8055 is an mTOR inhibitor that not only inhibits mTOR but is also an antagonist of PI3K. Herein, AZD8055 inhibits mTORC1 phosphorylation and mTORC2 substrates and has shown significant growth inhibition in xenografts across a broad range of tumor types. Currently, AZD8055 is in Phase I/II trials as a potential cancer treatment [156].

A wide variety of cancer cell lines including GBM cell lines have shown sensitivity to glutamine starvation. Studies researching the effect of enzymatically lowering blood glucose levels using L-asparaginase have shown success in glutamine depletion, but clinical treatment in adults has resulted in high levels of toxicity. More research must be conducted to determine the role of L-asparaginase in GBM treatment [157,158]. c-Myc promotion of glutamine uptake and catabolic processes makes targeting c-Myc-induced-glutamine addiction a potential subject for therapy. In a parallel fashion, targeting branched-chain amino acids (BCAAs) in the BCAA degradation pathway has also shown potential as well. Several enzymes have been identified as potential targets. For instance, BCAT1 exhibits a significantly elevated expression in GBM cells compared to normal cells [159]. Hence, using BCAT1 inhibitors has potential clinical implications. One study indicated curcumin as a BCAT1 inhibitor to induce apoptosis in tumor cells. Gabapentin was another clinically identified BCAT1 inhibitor as well. Other BCAA inhibitors are also being investigated as potential therapeutic agents in GBMs (Table 1) [26,166,177].

### 4.3. Lipid Metabolism

Targeting lipid metabolism has been investigated as a potential avenue to inhibit tumor growth and improve patient outcomes. These methods leverage the altered lipid metabolic pathways that are characteristic of cancer cells. Recent studies have highlighted the significance of enzymes in lipid metabolism, showing that inhibitors targeting enzymes like ACLY are highly effective in reducing lipid production in tumor cells, which often exhibit upregulated and more active lipid metabolism compared to normal cells. This increased activity meets the high demand for lipids needed for rapid tumor growth, supplying essential components for membrane formation and other cellular functions. By inhibiting ACLY, the synthesis of acetyl-CoA is disrupted, which in turn reduces the production of critical lipids necessary for rapid cell division (Table 1). Various studies have demonstrated that inhibitors such as hydroxycitrate and SB-204990 can effectively halt cancer cell growth [160]. Bempedoic acid, an approved drug targeting ACLY, reduces cholesterol synthesis and is administered as a prodrug. Once it enters the body, it is metabolized in the liver into its active coenzyme A (CoA) form, which directly inhibits ACLY [161]. Although primarily used to lower cholesterol, bempedoic acid’s ability to reduce lipid production suggests it may also have potential utility in cancer treatment in the future (Table 1).

Advances in understanding the key enzymes involved in the lipid metabolic pathways can improve outcomes for glioblastoma patients. By inhibiting FASN, the enzyme in lipid metabolism responsible for synthesizing palmitate from acetyl-CoA and malonyl-CoA using NADPH [34], combined with standard treatments might significantly weaken cancer cells (Table 1). This weakening makes them more vulnerable to the effects of chemotherapy and radiation, which damages the cellular structures of tumor cells. When these cells are deprived of fatty acids, they are less able to repair damage, leading to higher rates of apoptosis [163,167]. FASN inhibitors like cerulenin, C75, orlistat, C93, and particularly TVB-2640, which has shown promise in recent studies, disrupt lipid metabolism (Table 1). This disruption is crucial due to FASN’s overexpression in cancer cells, which leads to increased lipid synthesis and suppresses TNF-α. This suppression, in turn, decreases the activation of enzymes like sphingomyelinase that produce ceramide—a lipid molecule essential for initiating cell death [168]. Recent research findings have demonstrated the effectiveness of combining TVB-2640 with bevacizumab, an inhibitor of angiogenesis that inhibits the vascular endothelial growth factor (VEGF), which is a protein abundantly expressed by tumor cells. Bevacizumab functions by inhibiting the formation of blood vessels that supply tumors with essential nutrients and oxygen. This combination is currently in the second phase of study and has demonstrated improved treatment outcomes, including higher response rates and extended progression-free survival compared to using bevacizumab alone. The combined treatment achieved a 56% overall response rate (ORR), with 17% of patients experiencing complete tumor disappearance of their tumors and 39% showing significant tumor reduction [164]. The six-month progression-free survival (PFS6) rate of 31.4% indicates that approximately one-third of the patients did not experience tumor growth or worsening of the disease six months after starting treatment, which is almost double the rate observed with bevacizumab alone (16%). This suggests that TVB-2640 enhances the effectiveness of bevacizumab in delaying disease progression [164].

### 4.4. Nucleotide Metabolism

There is a wide variety of targets associated with the rewired pathways in nucleotide metabolism for GBMs. For instance, inhibitors of PI3K-related kinase (PIKK), such as ceralasertib, could potentially inhibit de novo purine synthesis in GBM cells (Table 1) [169]. In a similar fashion, CAD inhibitors are small molecules, such as PALA (N-phosphonacetyl-L-aspartate), targeting carbamoyl-phosphate synthetase 2, aspartate transcarbamylase, and dihydroorotase could be explored as potential inhibitors of pyrimidine synthesis in GBMs (Table 1) [170]. Additionally, DHODH inhibitors, like BAY2402234, have shown promise against GSCs and significant anti-tumor efficacy in mouse xenograft models of GBMs (Table 1) [54]. Targets for nucleotide salvage pathways include hypoxanthine-guanine phosphoribosyltransferase (HGPRT) inhibitors and TK inhibitors, such as erlotinib and gefitinib [171,172]. Additionally, dihydropyrimidine dehydrogenase (DPD) inhibitors, such as eniluracil, are potential targets for purine and pyrimidine catabolism that warrant further investigation in the context of GBMs (Table 1) [173].

As previously eluded, chemoresistance is a significant barrier in GBM treatment, specifically in the case of TMZ. Targeting nucleotide metabolism offers a potential alternative strategy to overcome this resistance in clinical applications. DHODH inhibitors could reduce the availability of nucleotides for DNA repair in resistant cells, thereby increasing sensitivity to TMZ. Additionally, targeting salvage pathways with HGPRT inhibitors could limit the tumor’s ability to recycle nucleotides, further sensitizing resistant cells [174].

### 4.5. TCA Cycle and Oxidative Phosphorylation

Therapeutic targets related to the TCA cycle and oxidative phosphorylation in GBMs include glutaminase, which catalyzes the conversion of glutamine to glutamate, providing α-KG for the TCA cycle [175] (Figure 2). Herein, inhibitors of glutaminase, such as CB-839, have been investigated as potential therapies to target glutamine addiction in cancer cells, including GBMs (Table 1) [176]. Additionally, α-KG dependence explains GBMs’ reliance on exogenous sources of α-KG to support TCA cycle function [64]. Hence, strategies aimed at limiting α-KG availability or disrupting its utilization could impair tumor growth. In regards to oxidative phosphorylation, electron transport chain (ETC) complex inhibitors, which target components of the mitochondrial ETC, such as complex I (e.g., metformin), complex II (e.g., sorafenib), and complex III (e.g., antimycin A), have been investigated for their potential to disrupt oxidative phosphorylation and induce cytotoxicity in cancer cells, including GBMs (Table 1) [149,178,179,180,181]. Furthermore, mitochondrial metabolism inhibitors are agents targeting mitochondrial metabolism, such as inhibitors of ATP synthase (e.g., oligomycin) or mitochondrial pyruvate carrier (e.g., UK-5099), and can impair ATP production and induce metabolic stress in cancer cells, potentially sensitizing them to other therapies (Table 1) [182,183]. In addition, small molecules targeting metabolic regulators implicated in oxidative phosphorylation dysfunction, such as AMP-activated protein kinase (AMPK) activators or sirtuin inhibitors, have been explored for their ability to modulate cellular metabolism and inhibit tumor growth in reprogrammed GBMs (Table 1) [184,185]. In the realm of T cell immunotherapy, glutarate has been shown to regulate T cell metabolism and differentiation [186].

In addition, devimistat is a therapeutic drug that targets pyruvate dehydrogenase and α-KG dehydrogenase. This drug is beneficial for attacking GBM cells that are dominantly reliant on oxidative metabolism. Devimistat has been shown to reduce TCA-cycle metabolites, induce cell death, and reduce proliferation in GBM cells (Table 1) [124,187]. Alternatively, imiprodones are a family of therapeutic drugs that influence the TCA cycle and cellular respiration (Table 1). These drugs promote the expression of TNF-related-apoptosis-inducing ligand (TRAIL). The mechanism by which TRAIL works involves death receptor 4 (DR4) and death receptor 5 (DR5). Not only do imiprodones induce TRAIL, but they also upregulate DR5 [124,188]. Studies reveal that there is a significant reduction in the oxygen consumption in GBM cells upon treatment with imiprodones. This indicates that a part of the offensive action of imipridones involves the suppression of cellular respiration and ATP-coupled respiration. This suppression of oxidative phosphorylation ultimately results in a lack of sufficient energy. Imipridone-mediated suppression of oxidative phosphorylation results in energy deprivation. The cellular stress brought upon by imipridones leads to the increased expression of transcription factors. An example of one of these transcription factors is ATF4, which is a transcription factor that is known to promote cellular survival under very hypoxic conditions. Imipridones also alter cellular metabolism by activation of caseinolytic mitochondrial matrix peptidase proteolytic subunit (CLPP). The drug binds and activates CLPP, which has negative effects on oxidative phosphorylation. Specifically, it incapacitates the complexes within the oxidative phosphorylation pathway, such as NDUFA12 (this protein is a part of complex I) as well as proteins related to both complexes IV and V (Table 1) [124].

## 5. Conclusions and Future Directions

The study of metabolic reprogramming specifically in GBM cells reveals the different metabolic pathways that are implicated in the resistance and progression of this tumor and the innovative therapeutic strategies that can target these adaptations. By understanding drivers of metabolic reprogramming, such as hypoxia, tumor microenvironment, and epigenetic factors, we have gained a deeper understanding of the complex and interconnected nature of these pathways. This knowledge not only highlights the resilience and adaptability of GBM cells but also paves the way for targeted therapeutic interventions. Exploring key metabolic pathways, including glycolysis (Warburg effect), amino acid, lipid, and nucleotide metabolism, has revealed several potential targets that could lead to more effective treatments.

Building on our current understanding of metabolic reprogramming in GBM cells, future directions should focus more on targeting the metabolic pathways that drive tumor cell proliferation and survival. The development and refinement of inhibitors against key metabolic regulators could lead to significant tumor reduction, as shown in many studies. Additionally, considering the complex interactions within the tumor microenvironment—particularly the role of hypoxia-inducible factors (HIFs) and the participation of immune cells like microglia and myeloid-derived suppressor cells—targeting these elements presents a viable strategy to counter the tumor-promoting conditions in GBMs. The integration of these metabolic inhibitors into combination therapies offers significant potential for overcoming the adaptive resistance often observed in GBM treatment. The use of such combination therapies might be most effective if applied in a preemptive manner depending on the mutational profile of the primary tumor rather than as a salvage avenue after the tumor recurs with potentially novel rewired metabolic pathways and more aggressive resistance strategies.

Furthermore, the potential of tunneling nanotubes in facilitating cellular communication and metabolic exchange within tumors represents a promising avenue for disrupting the cellular networks that sustain GBMs’ resilience and facilitate the spread of metabolic resistance mechanisms through sharing mitochondria or key enzymes. Advancing our understanding of these mechanisms could pave the way for innovative therapeutic approaches, leading to more effective treatment strategies. Ultimately, exploring these avenues can accelerate the translation of these findings into clinical settings, thereby improving patient outcomes.

## 6. Limitations

While this review provides a comprehensive overview of the current understanding of metabolic reprogramming in GBMs and emerging strategies to target it, several limitations must be acknowledged. First, many of the targeted therapies discussed are backed by evidence from preclinical studies, which poses a challenge, as these findings do not always equate to successful outcomes in the clinical setting. Additionally, the inherent complexity and variability of GBMs further complicate treatment development, as the therapeutic strategies discussed may lack the breadth needed to target all different subpopulations with a tumor, which have different metabolic profiles and rely on different strategies for adaptation. Hence, the potential of metabolic targeting conveyed in this review will not consolidate into a clinical reality except after it passes through rigorous testing in prospective randomized trials and proves effective in addressing real-life challenges seen in clinical settings.
